# Assessment of left ventricular dyssynchrony and cardiac function in patients with different pacing modes using real-time three-dimensional echocardiography: Comparison with tissue Doppler imaging

**DOI:** 10.3892/etm.2013.1292

**Published:** 2013-09-13

**Authors:** MIN DAI, JUAN LU, DA-JUN QIAN, JIAN-FENG CAI, XIAO-YU LIU, XIAO-QING WU, ZHEN-YU YANG, XIAO-RONG LI, RU-XING WANG

**Affiliations:** 1 Department of Cardiology, Affiliated Wuxi People’s Hospital of Nanjing Medical University, Wuxi, Jiangsu 214023;; 2 Department of Cardiology, Affiliated Jiangyin People’s Hospital of Medical College of Southeast University, Wuxi, Jiangsu 214400, P.R. China

**Keywords:** real-time three-dimensional echocardiography, tissue Doppler imaging, cardiac pacing, left ventricular dyssynchrony

## Abstract

The aim of this study was to evaluate the left ventricular mechanical dyssynchrony (LVMD) and left ventricular dysfunction of patients in AAI, DDD and VVI pacing modes using real-time three-dimensional echocardiography (RT3DE) and tissue Doppler imaging (TDI). The results from the RT3DE and TDI were subsequently compared. Twenty patients with sick sinus syndrome (SSS) who had undergone the implantation of a dual-chamber pacemaker were enrolled in this study and the pacemakers were programmed to AAI, DDD and VVI modes, sequentially. The RT3DE and TDI parameters were obtained following pacing for 24 h in each mode. With RT3DE, we measured the systolic dyssynchrony indices, including Tmsv_16_-SD%, Tmsv_12_-SD%, Tmsv_6_-SD%, Tmsv_16_-Dif%, Tmsv_12_-Dif% and Tmsv_6_-Dif%, left ventricular end-diastolic volume (LVEDV), left ventricular end-systolic volume (LVESV) and left ventricular ejection fraction (LVEF), respectively. With TDI, we measured the standard deviation and the maximal difference in time from the QRS onset to the peak systolic velocity for 12 left ventricular myocardial segments, i.e. Ts-SD and Ts-Dif, respectively. The results showed that the Tmsv_16_-SD% and Ts-SD in the AAI mode were significantly lower than those in the DDD and VVI modes (P<0.05); however, there were no significant differences between the DDD and VVI modes (P>0.05). The LVEF in the AAI, DDD and VVI modes was 63.1±8.9, 58.6±11.2 and 57.9±7.6%, respectively (P>0.05). There were negative correlations between the LVEF and Tmsv_16_-SD% (r, −0.651; P<0.001) and Ts-SD (r, −0.649; P<0.0001). A moderate correlation (r, 0.698; P<0.0001) was observed between Tmsv_16_-SD% and Ts-SD. The concordance rate between Tmsv_16_-SD% and Ts-SD for detecting LVMD was 76%. This study showed that DDD and VVI pacing modes induced significant LVMD and a reduction in LVEF, unlike the AAI pacing mode. RT3DE and TDI were capable of objectively evaluating LVMD; however, each method had certain faults. At present, there is a lack of a uniform standard for assessing LVMD; therefore, the use of a variety of techniques and indices is necessary in order to comprehensively evaluate LVMD in patients with different cardiac pacing modes.

## Introduction

The use of an optimal pacing mode for the treatment of bradycardia is important. In patients with sick sinus syndrome (SSS), DDD and VVI pacing modes may increase the risk of congestive heart failure, atrial fibrillation and thromboembolism (1–3). The main mechanism behind this may be that the electrical dyssynchrony induced by the abnormal ventricular activation site and sequence in the DDD and VVI pacing modes leads to left ventricular mechanical dyssynchrony (LVMD) and left ventricular (LV) dysfunction. Therefore, it is important that the LVMD and LV function is objectively estimated in patients with various pacing modes.

Echocardiography is important in assessing LVMD and LV function. At present, real-time three-dimensional echocardiography (RT3DE) and tissue Doppler imaging (TDI) are the most sensitive and commonly used techniques for the quantification of LVMD. The aim of the present study was to: i) evaluate LVMD and LV function in different pacing modes using RT3DE and TDI; and ii) compare dyssynchrony indices derived from RT3DE with different TDI indices when acquired from the same patients. To the best of our knowledge, this is the first time a study of this nature has been performed. The study may provide objective data to act as a foundation for clinical physicians when choosing optimal pacing modes.

## Materials and methods

### Study population

Twenty patients, including 12 males and 8 females (mean age, 58±11 years), with SSS and intact intrinsic atrioventricular (AV) conduction were enrolled during the period from August 2011 to December 2012. All patients received dual-chamber pacemaker implantation with the atrial leads placed in the right atrial appendage and the right ventricular leads positioned in the right ventricular apex. The patients had normal cardiac function [LV ejection fraction (LVEF), 50–72%], normal cardiac anatomy and no history of cardiovascular diseases. Any patients with atrial fibrillation, coronary heart disease, cardiomyopathy, AV conduction block and other rhythm disturbances were excluded. Pacemakers were provided by Medtronic (Minneapolis, MN, USA), Biotronik SE & Co. KG (Berlin, Germany) and St. Jude Medical, Inc.. (St. Paul, MN, USA). All the pacemakers were programmed with a basic rate of 70 bpm. The paced AV delay in the dual chamber pacing was programmed to 150±23 msec. All patients volunteered to participate in the study and were included once spoken and written informed consent had been received. The study protocol was approved by the Ethics Committee of the Affiliated Wuxi People’s Hospital of Nanjing Medical University (Wuxi, China).

### Pacemaker programming

Dual-chamber pacemakers were programmed for AAI, DDD and VVI pacing modes, respectively, i.e. the AAI pacing mode was initially programmed in all patients, prior to the pacemakers being programmed from AAI to DDD modes and then from DDD to VVI modes. Subsequent to pacing being performed in each mode for 24 h, the RT3DE and TDI images were acquired, respectively.

### RT3DE acquisition

RT3DE was performed using a commercially available echocardiography system (iE33; Philips Medical Systems, Andover, MA, USA) by employing an X3 matrix transducer. The individuals were asked to hold their breath and the images were coupled with an electrocardiographic record. The images were stored in the hard disk of the echocardiography system for further offline analysis with special software (QLAB, version 8.1; Philips Medical Systems) for the same equipment. The parameters evaluated using RT3DE included LV end-diastolic volume (LVEDV), LV end-systolic volume (LVESV), LVEF and RT3DE volume-time curves (VTCs). The left ventricle was divided into 17 segments, from apex to base, according to the segmentation schema of the American Heart Association and the American Society of Echocardiography (4), and the regional VTCs were obtained for each segment. To assess systolic dyssynchrony, the standard deviation (SD) of time from the QRS onset to the minimal systolic regional volume was obtained for 16 segments, i.e. 6 basal, 6 middle and 4 apical segments (Tmsv_16_-SD); as well as 12 segments, i.e. 6 basal and 6 middle segments (Tmsv_12_-SD), and the 6 basal segments (Tmsv_6_-SD) of the left ventricle in each patient. In addition to the Tmsv index, the maximal difference (Dif) in time from the QRS onset to the minimal regional systolic volume for the l6, 12 and 6 segments of the left ventricle (Tmsv_16_-Dif, Tmsv_12_-Dif and Tmsv_6_-Dif, respectively) was automatically calculated. All the systolic dyssynchrony indices were normalized as percentages of the RR-interval (Tmsv_16_-SD%, Tmsv_12_-SD%, Tmsv_6_-SD%, Tmsv_16_-Dif%, Tmsv_12_-Dif% and Tmsv_6_-Dif%) (5). The cut-off value of Tmsv_16_-SD% used in this study was 8.3% (6). The higher the Tmsv_16_-SD value, the worse the LV synchronicity.

### TDI acquisition

TDI echocardiograms were acquired using the iE33 echocardiography system with a broadband transducer (S5-1,2-5 MHz). The images were coupled with an electrocardiographic record. The TDI echocardiographic examinations were performed in accordance with the guidelines of the American Society of Echocardiography (7). The parameters evaluated using TDI were Ts-SD and Ts-Dif, which were defined as the standard deviation and the maximal difference in time, respectively, from the QRS onset to the peak systolic tissue velocity for 12 segments of the left ventricle, i.e. 6 basal and 6 middle segments obtained from two, three and four-chamber apical views. In addition, TDI analysis of 6 basal and 6 middle segments was obtained from two, three and four-chamber apical views. The cut-off value of Ts-SD used in this study was 32.6 msec (8,9). The higher this value, the worse the LV synchronicity. The peak speed of the early diastolic phase in the mitral valve annulus (E) was measured using pulsed Doppler. In addition, the peak speed of the early diastolic phase in the mitral valve annulus (Em) was measured using TDI and E/Em was calculated.

The RT3DE and TDI analysis of each patient was undertaken by three different observers and the data shown are the mean of three consecutive measurements.

### Statistical analysis

Continuous variables are presented as the mean ± SD. A one-way analysis of variance (ANOVA) test was used to evaluate the differences among the three modes and a Student-Newman-Keuls test was performed to make a comparison between two modes. Non-parametric tests were used if the data were abnormally distributed or showed heterogeneous variance. To evaluate the correlation analysis, Pearson’s correlation method (r) was employed and the χ^2^ test was used to compare categorical variables. Data were processed with a statistical analysis software (SPSS for Windows version 19.0; SPSS, Inc., Chicago, IL, USA). P<0.05 was considered to indicate a statistically significant difference.

## Results

### Evaluations of LVMD using RT3DE

**Figure 1.**
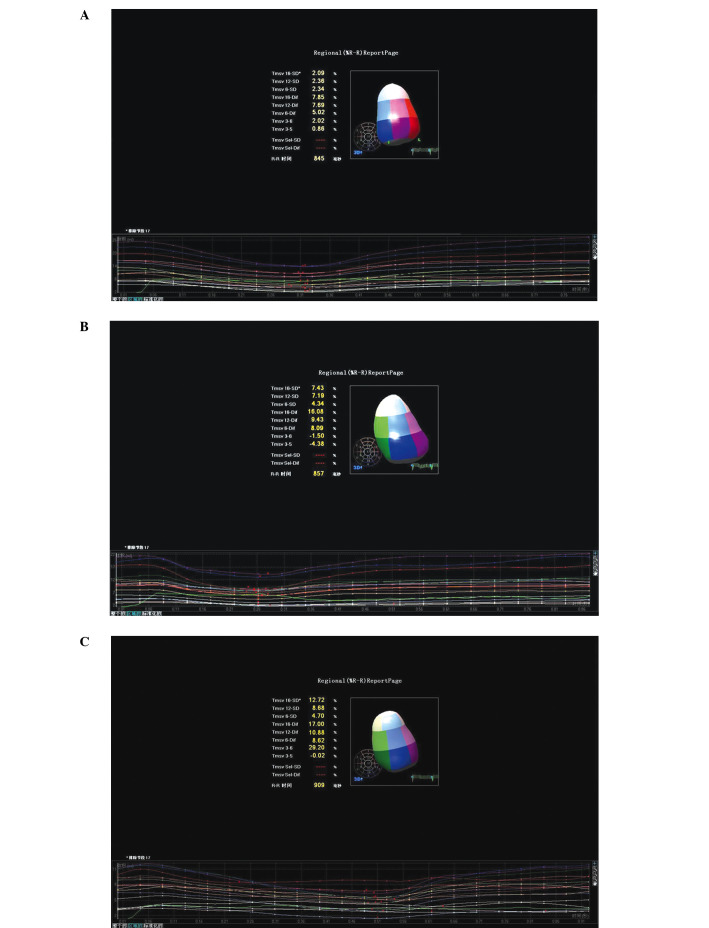
Real-time three-dimensional echocardiography (RT3DE) 17 regional left venrticular (LV) volume-time curves (VTCs) for one patient. (A) VTCs in one patient from the AAI pacing mode. The Tmsv_16_-SD% and Tmsv_16_-Dif% of LV dyssynchrony indices were 2.09 and 7.85%, respectively. (B) VTCs in the same patient from the DDD pacing mode. The Tmsv_16_-SD% and Tmsv_16_-Dif% of LV dyssynchrony indices were 7.43 and 16.08%, respectively. (C) VTCs in the same patient from the VVI pacing mode. The Tmsv_16_-SD% and Tmsv_16_-Dif% of LV dyssynchrony indices were 12.72 and 17.0%, respectively.

The descriptive analysis of RT3DE-derived LV dyssynchrony indices in patients with AAI, DDD and VVI pacing modes are shown in Table I. The Tmsv_16_-SD% (2.9±1.6) and Tmsv_16_-Dif% (5.8±2.6) in the AAI mode were significantly lower than those in the DDD mode (9.1±3.3%, P<0.05 and 12.8±6.2%, respectively; P<0.05) and the VVI mode (11.2±3.9%, P<0.05 and 15.6±5.3%, respectively; P<0.05). The same trends were also observed for Tmsv_12_-SD%, Tmsv_12_-Dif%, Tmsv_6_-SD% and Tmsv_6_-Dif%. These results showed that LV systolic synchronization in the AAI mode was superior to that in the DDD and VVI modes. However, no significant difference between the DDD and VVI modes was observed (P>0.05). The VTCs of the AAI, DDD and VVI pacing modes are shown in Fig. 1.

### Evaluations of LVMD using TDI

The descriptive analysis of TDI-derived LV dyssynchrony indices in patients with AAI, DDD and VVI pacing modes are shown in Table I. The Ts-SD in the AAI, DDD and VVI modes was 23.6±4.9, 42.3±9.7 and 46.1±5.6 msec, respectively, while the Ts-Dif in the AAI, DDD and VVI modes was 37.9±12.6, 106±23.6 and 112±28.7 msec, respectively. The Ts-SD and Ts-Dif values for the DDD and VVI modes were significantly different from those for the AAI mode (P<0.05). However, there was no significant difference between the DDD and VVI modes (P>0.05).

### LV systolic and diastolic function

**Figure 2.**
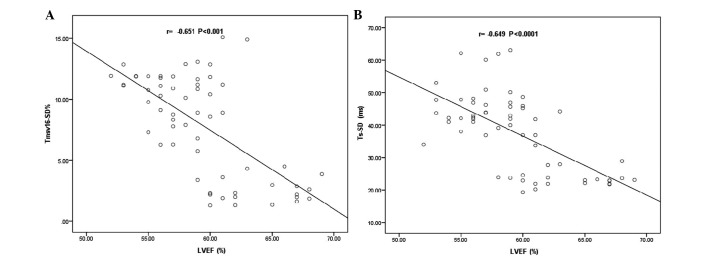
Correlation analysis between (A) Tmsv_16_-SD% and left ventricular ejection fraction (LVEF), and (B) Ts-SD and LVEF in the patient cohort. Tmsv, time from QRS onset to minimal systolic regional volume; SD, standard deviation; Ts, time from QRS onset to peak systolic tissue velocity.

The changes in LV systolic and diastolic function in patients with AAI, DDD and VVI pacing modes are shown in Table II. The LVEF in the AAI, DDD and VVI modes was 63.1±8.9, 58.6±11.2 and 57.9±7.6%, respectively (P>0.05). Pearson’s correlation analysis showed that the LVEF was inversely correlated with the RT3DE and TDI-derived LV dyssynchrony indices. The descriptive analysis is shown in Table III. The coefficient of correlation (r) between the LVEF and Tmsv_16_-SD% was −0.651 (P<0.001; Fig. 2A), while the correlation between the LVEF and Ts-SD was −0.649 (P<0.0001; Fig. 2B). No significant differences were identified in LVEDV, LVESV and E/Em among the AAI, DDD and VVI pacing modes (P>0.05).

### Correlation and concordance between RT3DE and TDI

**Figure 3.**
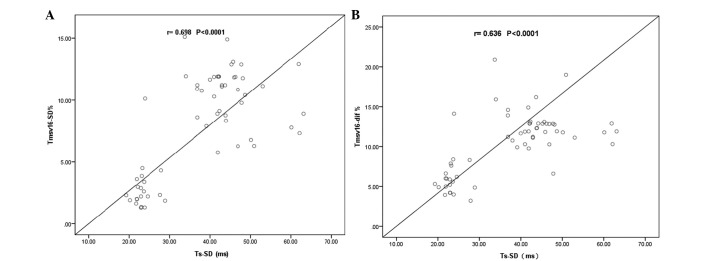
Correlation analysis between (A) Tmsv_16_-SD% and Ts-SD and (B) Tmsv_16_-Dif% and Ts-SD in the patient cohort. Tmsv, time from QRS onset to minimal systolic regional volume; SD, standard deviation; Ts, time from QRS onset to peak systolic tissue velocity; Dif, maximal difference.

The correlations between the RT3DE and TDI-derived LV dyssynchrony indices are shown in Table III. As shown in Fig. 3A, the correlation between Tmsv_16_-SD% and Ts-SD was 0.698 (P<0.0001), while the correlation between Tmsv_16_-Dif% and Ts-SD was 0.636 (P<0.0001, Fig. 3B). Moderate correlations were also observed between the other RT3DE and TDI-derived LV dyssynchrony indices (r, 0.509–0.639; P<0.05). Tmsv_16_-SD% and Ts-SD showed a concordance rate of 76% for detecting LVMD, with two variables being above the normal cutoff values in 34 cases, and two variables being below the normal cut-off values in 12 cases. In the remaining 14 cases, eight had only a Tmsv_16_-SD% above the normal cut-off value, while six had only a Ts-SD above the normal cut-off value.

## Discussion

Numerous studies have demonstrated that the use of DDD and VVI modes in the setting of right ventricular apical (RVA) pacing may lead to abnormal electrical activation and LV dyssynchrony (10–13). Cardiac pacing in various modes may induce AV and inter- and intraventricular dyssynchrony accordingly. Previous investigations have also shown LVMD to be correlated with LV remodeling, LV enlargement, LV dysfunction, poor hemodynamic outcome and the occurrence of major cardiac events (14,15).

At present, a number of different types of echocardiographic technologies are used as major methods for the evaluation of LVMD; however, there is no gold standard. In our prospective study, we attempted to quantify LV dyssynchrony and cardiac function in patients with AAI, DDD and VVI pacing modes using RT3DE, and compared the results from the RT3DE with the different dyssynchrony indices derived from TDI for the same patient. Previous studies have indicated that measuring the standard deviation and the maximal difference in time from the QRS onset to the peak systolic longitudinal velocity for 12 LV myocardial segments using TDI is useful for quantifying systolic dyssynchrony (9,16–20). However, the physics of TDI present certain limitations, such as the fact that TDI is not able to reflect the dyssynchrony of the entire left ventricle. This is due to TDI being unable to obtain information from the apical region, as a result of the angle dependency of TDI. In addition, tethering from adjacent ventricular segments may affect TDI measurements, and TDI is restricted to the analysis of the longitudinal wall motion of relatively few cardiac segments. By contrast, RT3DE presents a number of advantages over the TDI technique for assessing LVMD. It enables the observation of the overall left ventricle as an entire structure (16 or 17-segment model) in the same heart beat, leading to VTCs derived from this analysis. Furthermore, while RT3DE has been demonstrated to assess LVMD, it additionally appears to evaluate LV remodeling and function. Thus, LV remodeling (i.e. LV volumes) and synchronicity may be assessed in a single analysis.

In the present study, the RT3DE and TDI-derived dyssynchrony indices in the AAI mode were significantly lower than those in the DDD and VVI modes (P<0.05); however, there was no significant difference between the DDD and VVI modes (P>0.05). These results showed that LV synchronization in the AAI mode was significantly superior to that in the DDD and VVI modes. In the AAI pacing mode, normal AV, left-right inter- and intraventricular conduction sequences were maintained, which was consistent with physiological ventricular activation; therefore, the synchronization of the AAI pacing mode was normal. In the DDD pacing mode, while the AV conduction sequence was maintained, the normal inter- and intraventricular conduction sequences were lost due to RVA pacing, which induced systolic and diastolic mechanical dyssynchrony. With regard to the VVI pacing mode, normal inter- and intraventricular conduction sequences, in addition to the AV conduction sequence, were lost. Therefore, the dyssynchrony in the VVI mode was worse than that in the DDD mode, although our study revealed no significant differences between the two modes (P>0.05).

In addition to inducing LVMD, cardiac pacing may also cause LV dysfunction. By measuring LVEF, LVEDV and LVESV, RT3DE was used to estimate the changes in cardiac systolic function. In this study, the LVEF decreased in the DDD and VVI modes following pacing for 24 h; however, there were no significant differences among the AAI, DDD and VVI modes (P>0.05). Previous studies have demonstrated an acute reduction in the LVEF of 6–13% following the initiation of DDD and VVI pacing (21,22). In addition, we observed that the RT3DE and TDI-derived dyssynchrony indices increased significantly with the severity of the LV systolic dysfunction. The Tmsv_16_-SD% and Ts-SD were inversely correlated with LVEF (r, −0.651; P<0.001 and r, −0.649; P<0.0001), which is consistent with previous studies (23,24). These results all indicate that the degree of LV dyssynchrony adversely affected global LV systolic function.

In the current investigation, a moderate correlation between RT3DE and TDI-derived dyssynchrony indices was identified (r, 0.509–0.698; P<0.05). A possible explanation is that it is not possible to perform examinations in certain segments, such as the apex, with TDI whereas RT3DE enabled 16 segments of the whole left ventricle to be analyzed. Furthermore, TDI only studied longitudinal cardiac motion, while RT3DE was able to analyze the longitudinal, circumferential and radial cardiac mechanical motions. This result was controversial, taking previous studies into consideration. A number of studies have revealed contradictory results concerning RT3DE and TDI indices, ranging from poor (r, 0.11; P=not significant) (25,26) to good (r, 0.80; P<0.01) correlation (6,23,24,27–32). Furthermore, the concordance rate between Tmsv_16_-SD% and Ts-SD was 76% in the current study. A previous study showed that Ts-SD and Tmsv_16_-SD% had a concordance rate of 56.5% and when there was no agreement between the two indices, it was observed that Ts-SD was abnormal in 38% of patients and Tmsv_16_-SD% was abnormal in 8% (6). Another study showed the concordance rate to be 79% (27). However, it is difficult to compare the results from the various studies, since the studies used different selection criteria.

In conclusion, this study demonstrated that RT3DE and TDI were able to objectively and accurately evaluate LV function and LVMD in patients with various pacing modes and that LV systolic synchronicity in the AAI mode was superior to that in the DDD and VVI modes. The RT3DE and TDI-derived LV dyssynchrony indices increased with worsening LVEF. In addition, the RT3DE-derived dyssynchrony index Tmsv_16_-SD% had a high concordance rate with the well-established TDI-derived dyssynchrony index Ts-SD. In the same patient, there was variability in the incidence of LVMD depending on the echocardiographic method used. Due to the lack of gold standard for the evaluation of LVMD, two different echocardiographic dyssynchrony indices appear to provide complementary, rather than opposing, information regarding the presence of LVMD. In clinical applications, if the conditions permit, it is preferable for all pacemakers to be programmed in the AAI mode, in order to reduce LV ventricular dyssynchrony, heart dysfunction and complications and to improve the survival rates.

The present study had certain limitations. In particular, the study was a self-contrasted and was a single center study with a small sample size. A gold standard for the validation of the various dyssynchrony indices does not exist to date. It is necessary to note that the relatively low temporal and spatial resolution of RT3DE imaging may make the feasibility of this technique in the overall population limited. The current study only analyzed the changes in the different pacemaker modes in the acute phase; therefore, the acquisition of data from long-term follow-up studies is necessary. Furthermore, compared with the results from the RT3DE, only longitudinal cardiac motion was studied with TDI; it is possible that a stronger correlation may have been identified with other methods, such as circumferential or radial strain analysis using speckle tracking. In this study, the right ventricular leads were placed in the right ventricular apex; further discussion regarding the LVMD in patients with different pacing modes may be appropriate when the right ventricular leads are placed in the right ventricular outflow tract.

## Figures and Tables

**Table I.** Different dyssynchrony indices in AAI, DDD and VVI pacing modes.

Dyssynchrony indices	AAI mode (n=20)	DDD mode (n=20)	VVI mode (n=20)
Tmsv_16_-SD%	2.9±1.6	9.1±3.3^a^	11.2±3.9^a,b^
Tmsv_12_-SD%	2.7±0.9	7.5±2.6^a^	8.2±3.1^a^,^b^
Tmsv_6_-SD%	2.3±1.2	5.7±2.5^a^	6.3±2.8^a^,^b^
Tmsv_16_-Dif %	5.8±2.6	12.8±6.2^a^	15.6±5.3^a^,^b^
Tmsv_12_-Dif%	4.9±2.2	12.0±3.8^a^	13.9±5.1^a^,^b^
Tmsv_6_-Dif%	3.7±1.9	7.5±2.6^a^	8.2±5.2^a^,^b^
Ts-SD (msec)	23.6±4.9	42.3±9.7^a^	46.1±5.6^a^,^b^
Ts-Dif (msec)	37.9±12.6	106±23.6^a^	112±28.7^a^,^b^

Values are shown as the mean ± standard deviation (SD).

a P<0.05 compared with AAI;

b P>0.05 compared with DDD. Tmsv, time from QRS onset to minimal systolic regional volume; Ts, time from QRS onset to peak systolic tissue velocity; Dif, maximal difference.

**Table II.** Left ventricular systolic and diastolic function in AAI, DDD and VVI pacing modes.

Echocardiographic parameters	AAI mode (n=20)	DDD mode (n=20)	VVI mode (n=20)
LVEDV (ml)	99.7±8.3^a^	99.9±8.3^a^	98.1±7.7^a^
LVESV (ml)	36.8±5.1^b^	39.7±4.5^b^	43.2±4.6^b^
LVEF (%)	63.1±8.9^c^	58.6±11.2^c^	57.9±7.6^c^
E/Em	4.92±0.96^d^	5.82±0.74^d^	5.70±0.63^d^

Values are shown as the mean ± standard deviation.

a P>0.05;

b P>0.05;

c P>0.05;

d P>0.05. LVEDV, left ventricular end-diastolic volume; LVESV, left ventricular end-systolic volume; LVEF, left ventricular ejection fraction; E, the peak speed of early diastolic phase in the mitral valve annulus using pulsed Doppler; Em, the peak speed of early diastolic phase in the mitral valve annulus using tissue Doppler imaging.

**Table III.** Correlations of RT3DE- and TDI-derived LV dyssynchrony indices and LVEF.

RT3DE	Ts-SD (TDI)		Ts-Dif (TDI)		LVEF	
	r	P-value	r	P-value	r	P-value
Tmsv_16_-SD%	0.698	<0.0001	0.612	<0.001	−0.651	<0.001
Tmsv_12_-SD%	0.639	<0.001	0.586	<0.001	−0.632	<0.001
Tmsv_6_-SD%	0.368	ns	0.322	ns	−0.398	ns
Tmsv_16_-Dif%	0.636	<0.0001	0.532	<0.05	−0.614	<0.001
Tmsv_12_-Dif%	0.585	<0.001	0.509	<0.05	−0.594	<0.001
Tmsv_6_-Dif%	0.328	ns	0.296	ns	−0.382	ns
LVEF	−0.649	<0.0001	−0.579	<0.001	-	-

RT3DE, real-time three-dimensional echocardiography; TDI, tissue Doppler imaging; LV, left ventricular; ns, no significance; Tmsv, time from QRS onset to minimal systolic regional volume; SD, standard deviation; Dif, maximal difference; LVEF, left ventricular ejection fraction.

## References

[1] Nielsen JC, Kristensen L, Andersen HR, Mortensen PT, Pedersen OL, Pedersen AK (2003). A randomized comparison of atrial and dual-chamber pacing in 177 consecutive patients with sick sinus syndrome: echocardiographic and clinical outcome. J Am Coll Cardiol.

[2] Sweeney MO, Hellkamp AS, Ellenbogen KA, Greenspon AJ, Freedman RA, Lee KL, Lamas GA, MOde Selection Trial (MOST) Investigators (2003). Adverse effect of ventricular pacing on heart failure and atrial fibrillation among patients with normal baseline QRS duration in a clinical trial of pacemaker therapy for sinus node dysfunction. Circulation.

[3] Wilkoff BL, Cook JR, Epstein AE, Greene HL, Hallstrom AP, Hsia H, Kutalek SP, Sharma A, Dual Chamber and VVI Implantable Defibrillator Trial Investigators (2002). Dual-chamber pacing or ventricular backup pacing in patients with an implantable defibrillator: the Dual Chamber and VVI implantable Defibrillator (DAVID) Trial. JAMA.

[4] Cerqueira MD, Weissman NJ, Dilsizian V, Jacobs AK, Kaul S, Laskey WK, Pennell DJ, Rumberger JA, Ryan T, Verani MS, American Heart Association Writing Group on myocardial Segmentation and Registration for Cardiac Imaging (2002). Standardized myocardial segmentation and nomenclature for tomographic imaging of the heart. A statement for healthcare professionals from the Cardiac Imaging Committee of the Council on Clinical Cardiology of the American Heart Association. Circulation.

[5] Horstman JA, Monaghan MJ, Gill EA (2007). Intraventricular dyssynchrony assessment by real-time three-dimensional echocardiography. Cardiol Clin.

[6] Kapetanakis S, Kearney MT, Siva A, Gall N, Cooklin M, Monaghan MJ (2005). Real-time three-dimensional echocardiography: a novel technique to quantify global left ventricular mechanical dyssynchrony. Circulation.

[7] Schiller NB, Shah PM, Crawford M, DeMaria A, Devereux R, Feigenbaum H, Gutgesell H, Reichek N, Sahn D, Schnittger I, American Society of Echocardiography Committee on Standards, Subcommittee on Quantitation of Two-Dimensional Echocardiograms (1989). Recommendations for quantitation of the left ventricle by two-dimensional echocardiography. J Am Soc Echocardiogr.

[8] Yu CM, Fung WH, Lin H, Zhang Q, Sanderson JE, Lau CP (2003). Predictors of left ventricular reverse remodeling after cardiac resynchronization therapy for heart failure secondary to idiopathic dilated or ischemic cardiomyopathy. Am J Cardiol.

[9] Yu CM, Fung JW, Zhang Q, Chan CK, Chan YS, Lin H, Kum LC, Kong SL, Zhang Y, Sanderson JE (2004). Tissue Doppler imaging is superior to strain rate imaging and postsystolic shortening on the prediction of reverse remodeling in both ischemic and nonischemic heart failure after cardiac resynchronization therapy. Circulation.

[10] van Oosterhout MF, Prinzen FW, Arts T, Schreuder JJ, Vanagt WY, Cleutjens JP, Reneman RS (1998). Asynchronous electrical activation induces asymmetrical hypertrophy of the left ventricular wall. Circulation.

[11] Prinzen FW, Hunter WC, Wyman BT, McVeigh ER (1999). Mapping of regional myocardial strain and work during ventricular pacing: experimental study using magnetic resonance imaging tagging. J Am Coll Cardiol.

[12] Wyman BT, Hunter WC, Prinzen FW, Faris OP, McVeigh ER (2002). Effects of single- and biventricular pacing on temporal and spatial dynamics of ventricular contraction. Am J Physiol Heart Circ Physiol.

[13] Wyman BT, Hunter WC, Prinzen FW, McVeigh ER (1999). Mapping propagation of mechanical activation in the paced heart with MRI tagging. Am J Physiol Heart Circ Physiol.

[14] Bader H, Garrigue S, Lafitte S, Reuter S, Jaïs P, Haïssaguerre M, Bonnet J, Clementy J, Roudaut R (2004). Intra-left ventricular electromechanical asynchrony. A new independent predictor of severe cardiac events in heart failure patients. J Am Coll Cardiol.

[15] Fauchier L, Marie O, Casset-Senon D, Babuty D, Cosnay P, Fauchier JP (2002). Interventricular and intraventricular dyssynchrony in idiopathic dilated cardiomyopathy: a prognostic study with fourier phase analysis of radionuclide angioscintigraphy. J Am Coll Cardiol.

[16] Yu C, Chau E, Sanderson JE, Fan K, Tang MO, Fung WH, Lin H, Kong SL, Lam YM, Hill MR, Lau CP (2002). Tissue Doppler echocardiographic evidence of reverse remodeling and improved synchronicity by simultaneously delaying regional contraction after biventricular pacing therapy in heart failure. Circulation.

[17] Bax JJ, Bleeker GB, Marwick TH, Molhoek SG, Boersma E, Steendijk P, van der Wall EE, Schalij MJ (2004). Left ventricular dyssynchrony predicts response and prognosis after cardiac resynchronization therapy. J Am Coll Cardiol.

[18] Søgaard P, Egeblad H, Kim WY, Jensen HK, Pedersen AK, Kristensen BØ, Mortensen PT (2002). Tissue Doppler imaging predicts improved systolic performance and reversed left ventricular remodeling during long-term cardiac resynchronization therapy. J Am Coll Cardiol.

[19] Bax JJ, Marwick TH, Molhoek SG, Bleeker GB, van Erven L, Boersma E, Steendijk P, van der Wall EE, Schalij MJ (2003). Left ventricular dyssynchrony predicts benefit of cardiac resynchronization therapy in patients with end-stage heart failure before pacemaker implantation. Am J Cardiol.

[20] Ansalone G, Giannantoni P, Ricci R, Trambaiolo P, Laurenti A, Fedele F, Santini M (2001). Doppler myocardial imaging in patients with heart failure receiving biventricular pacing treatment. Am Heart J.

[21] Nahlawi M, Waligora M, Spies SM, Bonow RO, Kadish AH, Goldberger JJ (2004). Left ventricular function during and after right ventricular pacing. J Am Coll Cardiol.

[22] Rosenqvist M, Isaaz K, Botvinick EH, Dae MW, Cockrell J, Abbott JA, Schiller NB, Griffin JC (1991). Relative importance of activation sequence compared to atrioventricular synchrony in left ventricular function. Am J Cardiol.

[23] Zhang Q, Yu C, Fung J, Zhang Y, Chan Y, Chan H, Yip GW, Sanderson JE (2005). Assessment of the effect of cardiac resynchronization therapy on intraventricular mechanical synchronicity by regional volumetric changes. Am J Cardiol.

[24] Park SM, Kim KC, Jeon MJ, Lee CK, Kim DH, Park KS, Lee WH, Kwan J (2007). Assessment of left ventricular asynchrony using volume-time curves of 16 segments by real-time 3 dimensional echocardiography: Comparison with tissue Doppler imaging. Eur J Heart Fail.

[25] Burgess MI, Jenkins C, Chan J, Marwick TH (2007). Measurement of left ventricular dyssynchrony in patients with ischaemic cardiomyopathy: a comparison of real-time three-dimensional and tissue Doppler echocardiography. Heart.

[26] Samir R, Tawfik M, EI Missiri AM, EI Shahid G, Maaty MA, EI Sayed M (2012). Assessment of left ventricular mechanical dyssynchrony using real-time three-dimensional echocardiography: a comparative study to Doppler tissue imaging. Echocardiography.

[27] Takeuchi M, Jacobs A, Sugeng L, Nishikage T, Nakai H, Weinert L (2007). Assessment of left ventricular dyssynchrony with real-time 3-dimensional echocardiography: comparison with Doppler tissue imaging. J Am Soc Echocardiogr.

[28] Soliman OI, van Dalen BM, Nemes A, Zwaan HB, Vletter WB, ten Cate FJ, Theuns DA, Jordaens LJ, Geleijnse ML (2009). Quantification of left ventricular systolic dyssynchrony by real-time three-dimensional echocardiography. J Am Soc Echocardiogr.

[29] Marsan NA, Bleeker GB, Ypenburg C, Ghio S, van de Veire NR, Holman ER, van der Wall EE, Tavazzi L, Schalij MJ, Bax JJ (2008). Real-time three-dimensional echocardiography permits quantification of left ventricular mechanical dyssynchrony and predicts acute response to cardiac resynchronization therapy. J Cardiovasc Electrophysiol.

[30] Faletra FF, Conca C, Klersy C, Klimusina J, Regoli F, Mantovani A, Pasotti E, Pedrazzini GB, De Castro S, Moccetti T, Auricchio A (2009). Comparison of eight echocardiographic methods for determining the prevalence of mechanical dyssynchrony and site of latest mechanical contraction in patients scheduled for cardiac resynchronization therapy. Am J Cardiol.

[31] van Dijk J, Dijkmans PA, Gotte MJ, Spreeuwenberg MD, Visser CA, Kamp O (2008). Evaluation of global left ventricular function and mechanical dyssynchrony in patients with an asymptomatic left bundle branch block: a real-time 3D echocardiography study. Eur J Echocardiogr.

[32] Raedle-Hurst TM, Mueller M, Rentzsch A, Schaefers HJ, Herrmann E, Abdul-Khaliq H (2009). Assessment of left ventricular dyssynchrony and function using real-time 3-dimensional echocardiography in patients with congenital right heart disease. Am Heart J.

